# Spatial Extension of Road Traffic Sensor Data with Artificial Neural Networks

**DOI:** 10.3390/s18082640

**Published:** 2018-08-12

**Authors:** Mariano Gallo, Giuseppina De Luca

**Affiliations:** Dipartimento di Ingegneria, Università del Sannio, piazza Roma 21, 82100 Benevento, Italy; pideluca@unisannio.it

**Keywords:** traffic sensors, smart roads, artificial neural networks, ITS

## Abstract

This paper proposes a method for estimating traffic flows on some links of a road network knowing the data on other links that are monitored with sensors. In this way, it is possible to obtain more information on traffic conditions without increasing the number of monitored links. The proposed method is based on artificial neural networks (ANNs), wherein the input data are the traffic flows on some monitored road links and the output data are the traffic flows on some unmonitored links. We have implemented and tested several single-layer feed-forward ANNs that differ in the number of neurons and the method of generating datasets for training. The proposed ANNs were trained with a supervised learning approach where input and output example datasets were generated through traffic simulation techniques. The proposed method was tested on a real-scale network and gave very good results if the travel demand patterns were known and used for generating example datasets, and promising results if the demand patterns were not considered in the procedure. Numerical results have underlined that the ANNs with few neurons were more effective than the ones with many neurons in this specific problem.

## 1. Introduction

The real-time knowledge of traffic flows is essential for implementing any control strategy on road networks, both in urban and rural contexts. All Intelligent Transportation Systems (ITSs) cannot do without traffic monitoring systems, which have to survey traffic data and transmit them to a control room and a centralised informatics system to allow the implementation of traffic control strategies.

A road traffic monitoring system is based on sensors, opportunely located on the network, which measure some features of traffic, mainly road flows (veh/h), densities (veh/km), and speeds (km/h). These sensors, especially in urban contexts where the networks are significantly dense, cannot be provided on all road links of the network, but only on some of them; usually, the greater the number of monitored links, the more the potential accuracy of traffic forecasting and, then, the effectiveness of control strategies. The cost of an ITS depends significantly on the number of monitored links; even if the costs of traffic sensors have decreased in the last decade, the trade-off between the number of sensors and the reliability of the monitoring system is an important issue to be considered.

In this paper, we propose a methodology for spatially extending traffic data obtained on monitored links to other links of the network in order to increase the quality of the whole system, limiting its cost. More in detail, we propose to use artificial neural networks (ANNs) for reaching this result; the ANN, once trained, will estimate traffic flows on unmonitored links starting from the flows on monitored links. To the best of our knowledge, this approach was studied only in a preliminary way and on a small network [1]; ANNs, instead, have been widely used for short-term traffic flow forecasting, as will be described in the next section.

The remainder of the paper is structured as follows: Section 2 explores the background of the problem, focusing on sensors, monitoring systems, and main applications of ANNs in the transportation sector, with particular attention to traffic flow forecasting. Section 3 describes the proposed approach, which is tested on a real-scale network in Section 4. Finally, Section 5 concludes the paper.

## 2. Background

### 2.1. Road Traffic Monitoring Systems and Technologies

In the literature, road traffic monitoring systems and technologies have been widely studied from different points of view. Some books and papers focused on technological aspects of sensors while others dealt more with sensor networks and their applications on ITSs; a comprehensive review would deserve a separate paper.

Manuals by Federal Highway Administration (FHWA) [2,3] reviewed many kinds of traffic sensors, exploring their technology, applications, installation techniques, maintenance, etc. Bennet et al. [4] summarised data collection technologies for road management, including traffic sensors. The technical note by Leduc [5] summarised methods for collecting road traffic data, as well. Salvo et al. [6] and Li et al. [7] studied the use of UAVs (unmanned aerial vehicles) for traffic monitoring.

An interesting survey about sensor networks was reported by Chong and Kumar [8] that, among others, identified traffic control as one of the main fields where these technologies could be used profitably, particularly, the wireless sensor networks. They also wrote: “*However, these sensors and the communication network that connect them are costly; thus, traffic monitoring is generally limited to a few critical points*”. Notwithstanding that the costs have recently decreased, even today only some ”critical” links are monitored, in the best hypothesis.

Tubaishat et al. [9] have surveyed existing wireless sensor network technologies for ITSs by identifying the following main applications: (a) monitoring parking lots; (b) traffic monitoring and control; and (c) traffic estimation. Regarding traffic estimation, they have identified two main problems to solve: (c.1) estimating traffic at unobserved locations; and (c.2) predicting future traffic. For solving problem (c.1), a learning-based method was proposed by Guitton et al. [10], using the Pearson correlation coefficient. Two other methods were proposed by Tubaishat et al. [9]: a statistical method based on Gaussian models and a method based on origin-destination matrix estimation. Problem (c.2), instead, has been more widely studied in the literature [11,12]; for this problem, ANNs have also been extensively adopted, as reported in the next subsection. Another review of wireless sensor networks applied to ITSs is proposed by Sharma et al. [13]. Some interesting technological aspects for managing the data centres in real-time that can be used in ITSs can be found in [14,15,16].

Some papers focused on “mobile” sensors; Sun et al. [17] studied the application of optical sensors on on-road vehicles, while D’Acierno et al. [18] proposed to use urban buses as probe vehicles for estimating urban traffic conditions. Floating car data were used in [19] for improving network performance with individual in-car routing advice and in [20] for estimating the origin and destination of trips.

### 2.2. ANNs and Their Application to Traffic Flow Forecasting

Artificial neural networks (ANNs) are a proven approach for predicting or reproducing several physical phenomena in different scientific fields; they attempt to emulate the learning mechanisms that characterise the human brain. ANNs were introduced in the 1940s in [21,22], but the first actual prototype using the perceptrons and the learning mechanism was proposed in [23]. The works by Minsky and Papert [24], Kohonen [25], Grossberg [26], Minsky [27], and Hopfield [28] contributed to the dissemination of the method and its mathematical formalisation.

The literature on ANNs is extensive. Some comprehensive books, besides the ones already quoted, are [29,30,31,32,33,34]. Moreover, many surveys can be found. A review of leading software for training and applying ANNs can be found in [35]. A review of methods for using feed-forward neural networks can be found in [36]. Random neural networks have been reviewed in [37]. A review of the trends in extreme learning machines (ELM) has been proposed in [38], where the ELM is an innovative approach for training the neural networks. Surveys of deep neural network architectures have been proposed in [39,40]. A review of evolutionary ANNs has been proposed in [41].

The ANN is considered as a black box model: the functions that link input and output data are not known and the relationships between them are hidden and not interpretable; therefore, this method can only be used if it is not necessary to explicitly know the form of the function. ANNs are also used when the relations between the input and output data are difficult to identify or formulate. Indeed, the ANNs are able to approximate the function without explicit assumptions on the relations between variables and on the functional form. The strength of the approach is that the ANN, once trained, can give results (estimated data) in a very short time (in practice, immediately), proving suitable for real-time applications. Weaknesses of ANNs are the following: the model is not extendible to other cases, even if similar, and is valid only insofar as the boundary conditions do not change much.

ANNs have been widely used in the transportation sector since the black box approach is not an actual problem for several applications; for instance, the spatial extension that we propose in this paper does not require explicitly identifying the relationships between inputs (flows on monitored links) and outputs (flows on unmonitored links).

The main works in the transportation field published before 1995 have been reviewed by Dougherty [42] that identified 13 main subject areas: (1) driver behaviour/autonomous vehicles; (2) parameter estimation; (3) pavement maintenance; (4) vehicle detection/classification; (5) traffic pattern analysis; (6) freight operations; (7) traffic forecasting; (8) transport policy and economics; (9) air transport; (10) maritime transport; (11) submarine vehicles; (12) metro operation; and (13) traffic control. A literature review on ANNs applied to traffic flow forecasting can be found in [43].

Recently, ANNs and their variants have been used for traffic sign recognition, which is an essential task in many applications, such as traffic surveillance and autonomous driving; in this context, deep neural networks have been proposed in [44,45]. Applications of ANNs to the shortest path problem can be found in [46,47], while the automated traffic incident detection was studied in [48]. Tanprasert et al. [49] proposed to use ANNs for driver identification.

Focusing on traffic forecasting, which is the object of our paper, numerous papers in the literature can be found; two literature reviews regard short-term forecasting [11,12], which has been widely studied with the ANNs. Indeed, this is a problem for which the black box approach is not an obstacle: we do not need to export the model to other cases or to know the relationships between inputs and outputs explicitly. The applicability of ANNs to such problems was studied by Kirby et al. [50], while numerous studies focused on applications of short-term forecasting to highways [51,52,53,54,55,56,57,58,59], urban contexts [60,61,62,63,64,65], or urban freeways [66]. Papers by Lin et al. [67] and Zheng Zhu et al. [68] considered, besides the short-term forecasting, a spatial extension of data. Another topic regards the origin-destination matrix estimation with ANNs, for which we refer to [69,70,71,72,73].

The examination of the literature has underlined that the spatial extension of road traffic data has not been sufficiently studied, even if traffic flow estimation and forecasting are identified as crucial problems for simulating road networks and implementing traffic control and management strategies. Moreover, the ANN appears to be a methodology that can be usefully adopted for solving the problem studied in this paper.

## 3. Proposed Approach

We propose to use ANNs for spatially extending traffic flow data. We assume that some links of a road network are monitored with proper sensors and other links, often a large percentage, are not monitored. The objective of the ANN is to forecast traffic flows on unmonitored links starting from the data collected on monitored links. The same result can be obtained using the estimation of origin-destination matrix from traffic counts [74,75] as follows: (1) traffic flow data collection on the monitored links; (2) correction of the OD matrix using the collected data; and (3) assignment of the OD matrix to the network model for estimating the traffic flows on all links of the network. This second approach requires high computing times due to the OD matrix correction and traffic assignment procedures; with a trained ANN, instead, the computing times are very short, as will be shown in Section 4. Moreover, there is a broad debate on the (un)reliability of the OD matrix correction procedure [76].

**Figure 1:**
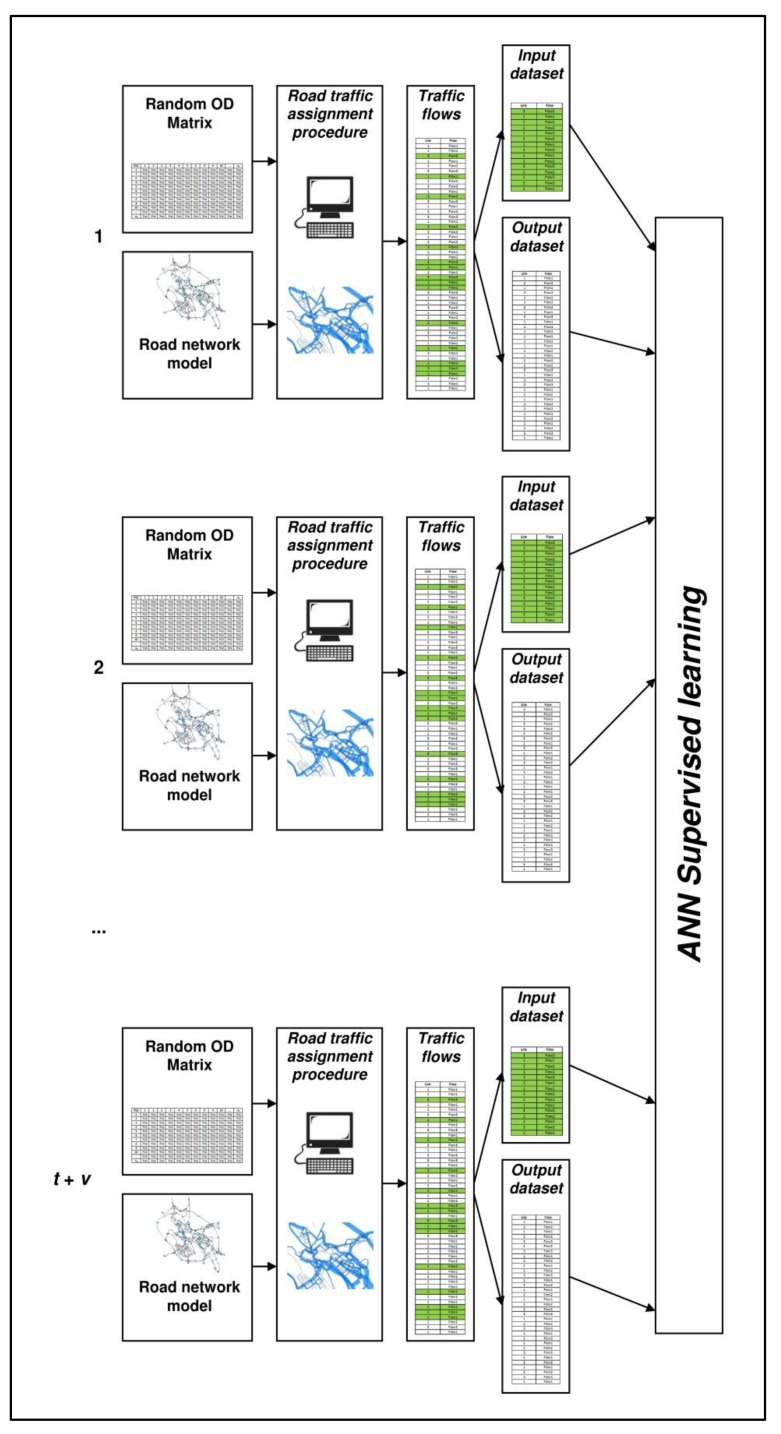
Generation of training datasets.

The crucial points of the method are the training of the ANN and how to verify if the approach works in terms of traffic flow forecasts. We use a supervised learning approach, wherein the example datasets are generated through simulation with the following procedure (see Figure 1):

- we build a road network model of the case study, as detailed as possible;
- we identify on this network the monitored links, *ml*, and the unmonitored links, *ul*;
- we randomly generate several origin-destination (OD) matrices, ***d****^i^* with *i* = 1, ..., *n*;
- we assign each matrix, ***d****^i^*, to the road network model, so as to generate the corresponding traffic flows on monitored links, ***f****^i^_ml_*, and on unmonitored links, ***f****^i^_ul_* with *i* = 1, ..., *n*; we assume that they are the real flows on the network and constitute the datasets for training, validating, and testing the ANN; specifically, input datasets contain ***f****^i^_ml_* and output datasets contain ***f****^i^_ul_*; and
- we divide the datasets into three groups: training sets (*i* = 1, ..., *t*), validation sets (*i* = *t* + 1, ..., *t* + *v*), and testing sets (*i* = *t* + *v* + 1, ..., *t* + *v* + *p*; *t* + *v* + *p* = *n*); first, two groups are used in the training procedure while the third group is used for evaluating the forecast capacity of trained ANNs.

Moreover, two critical issues to consider are the design of the ANN and the random method for generating the OD matrices. Indeed, theoretically, infinite configurations of ANNs can be proposed for solving the same problem. There are different kinds of ANNs, such as single or multilayer feed-forward networks and recurrent networks. Focusing on feed-forward networks, they can be structured considering a different number of layers and neurons for each layer; hence, in general, it is necessary to test different configurations so as to find the best one for the specific problem. About this problem, recently Philipp and Carbonell [77] have proposed a method for automatically determining the optimal size of a neural network.

Regarding the random generation of OD matrices, we propose two different methods. The first one randomly generates each OD matrix cell value between zero and a maximum value that can be chosen in the function of information about travel demand. The second method randomly generates each OD matrix cell value between a minimum and a maximum value that can be different for each cell; we can adopt this approach only if we have an estimate of the real OD matrix. In most cases, these estimated matrices are available from traffic analyses conducted in the same study area where the monitoring system has to be implemented. Indeed, since the travel demand follows some patterns (e.g., the demand between some origins and destinations is high, while between others it is low), to consider them in the proposed approach should improve the results. In this case, different ANNs have to be trained with reference to different periods (morning peak-hour, afternoon peak-hour, off-peak hour and so on). In the following, we will test both methods for obtaining the OD matrices used for generating the example datasets and four different single-layer ANNs, differing in the number of neurons.

## 4. Numerical Results

**Figure 2:**
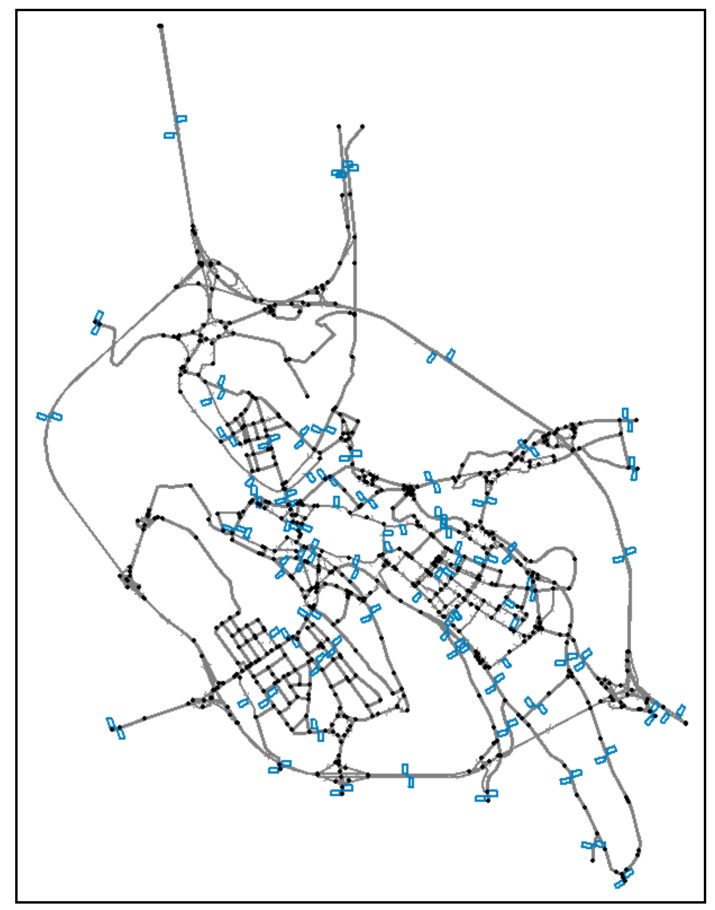
Road network model and monitored links.

The case study is based on the road network of Benevento, which is a city with about 61,000 inhabitants in the south of Italy. The road network model (see Figure 2) is very detailed and is composed of 949 road segments (1577 oriented links), 678 nodes, and 80 centroids (OD matrices with 6400 OD pairs); centroids represent in the model the points of origin and the destination of trips between zones. This network model represents 216 km of roads. All elements are modelled considering their features, such as length, speed, and capacity of the links and regulation methods (traffic light, stop, give way, roundabout, etc.) of nodes. Moreover, three OD matrices were available in the following periods: morning peak-hour (MP), afternoon peak-hour (AP), and off-peak-hour (OP); these matrices have been estimated by using mathematical models and road traffic surveys during the drafting of the Urban Traffic Plan of Benevento. Specifically, the traffic surveys were performed in the 139 road sections reported in Figure 1; in our tests, we assume that the traffic monitoring system provides sensors in these road sections: the corresponding links represent, therefore, the monitored links. Other real links of the network, instead, will be considered the unmonitored links. The data on road traffic flows were pre-processed, eliminating from the set of unmonitored links the ones with null traffic flows; we need to remove these links since the road network model is sometimes unable to estimate low traffic flows on local roads and it could distort the results. Then, in our problem, we consider 139 monitored links and 1186 unmonitored links.

In this case study, we have tested several ANNs, trained with different datasets. The first set of training data was obtained starting from OD matrices randomly generated, choosing a maximum value of the OD flow, without considering the patterns of the travel demand (same maximum value for all OD pairs). This maximum value is different in the different periods (MP, AP, and OP). We have to use this approach when the estimations of the OD matrices are not available. The second training dataset, instead, was generated by perturbing each OD pair demand flow, multiplying its estimated value by a random number between 0.8 and 1.2; this approach is possible only if we have the estimation of the OD matrix of each period (MP, AP, and OP). In this way, the patterns of the demand are taken explicitly into account.

Overall, we generated 180 matrices, with the first method (without considering the demand pattern), and 600 matrices, 200 in each period (MP, AP, and OP), with the second method (considering the demand pattern); each matrix was assigned to the road network so as to generate a traffic flow dataset. In the first case, 150 traffic flow datasets were used for training the ANN, while the others were used for verifying the quality of the results. In the second case, 190 traffic flow datasets were used for training the ANN in the corresponding period, and the others for the verification. Finally, 570 traffic flow datasets (190 in each period) were used for training an ANN in the second case, considering the three periods at the same time. We trained several ANNs in each case; all ANNs were feed-forward with a single layer, but with different numbers of neurons: 6, 10, 20, and 50. Table 1 summarises all 20 trained ANNs.

To evaluate the capacity of the ANNs to estimate road traffic flows on unmonitored links we have used three forecast error measures: mean square error (*MSE*), root mean square error (*RMSE*), and relative root mean square error (*RMSE%*). *RMSE%* allows for comparing the results corresponding to different demand levels and, then, is used to identify the best and the worst cases. In our tests, these measures are calculated as follows:

*MSE* = (1/*n_ul_*)⋅Σ*_j_*_ = 1, ..., *n*_*_ul_* (*f_j_^sim^* − *f_j_^ANN^*)^2^

*RMSE* = (*MSE*)^1/2^

*RMSE%* = *RMSE*/(Σ_*j* = 1, ..., *n_ul_*_*f_j_^ANN^*/*n_ul_*)

where *n_ul_* is the number of unmonitored links, *f_j_^sim^* is the traffic flow on link *j* obtained with simulation (the real flow, under our assumptions), and *f_j_^ANN^* is the traffic flow estimated with the trained ANN.

Moreover, we have calculated the coefficient of determination, *R*^2^, as follows:

*R*^2^ = 1 − (Σ*_j_*_ = 1, ..., *n*_*_ul_* (*f_j_^sim^* − *f_j_^ANN^*)^2^/Σ*_j_*_ = 1, ..., *n*_*_ul_* (*f_j_^sim^* − *M*(*f^sim^*))^2^)

where *M*(*f^sim^*) is the mean of the simulated traffic flows. The coefficient of determination is used for evaluating the capacity of a model to replicate the observed data; the maximum value of *R*^2^ is 1 (perfect capacity of the model to reproduce the observed data: *f_j_^sim^* = *f_j_^ANN^* on each link *j*) and the closer *R*^2^ to 1, the higher the goodness of the calibrated model. Even if, in our case, we lack an explicit model, we can use the coefficient of determination for measuring the capacity of the ANN to reproduce the simulated flows.

Naturally, all of these measures were calculated with datasets that were not used in the phase of training. In Table 2 we summarise the main results, by reporting for each ANN the best and the worst cases. In Table 3 we report the epochs and the computing times for training the ANNs; it can be noted that the training procedure is fast, requiring from some seconds up to 1 min and 37 s. The use of the trained ANNs for estimating traffic flows requires, instead, less than a second in all cases.

**Figure 3:**
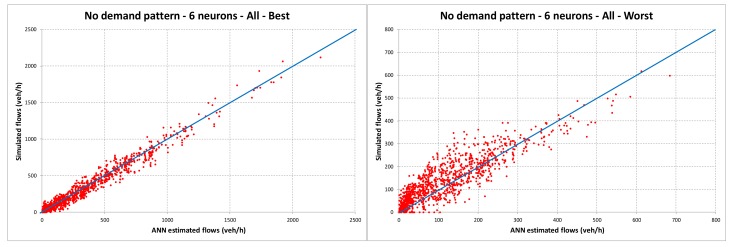
Dispersion diagrams without the demand pattern for the ANN with six neurons.

**Figure 4:**
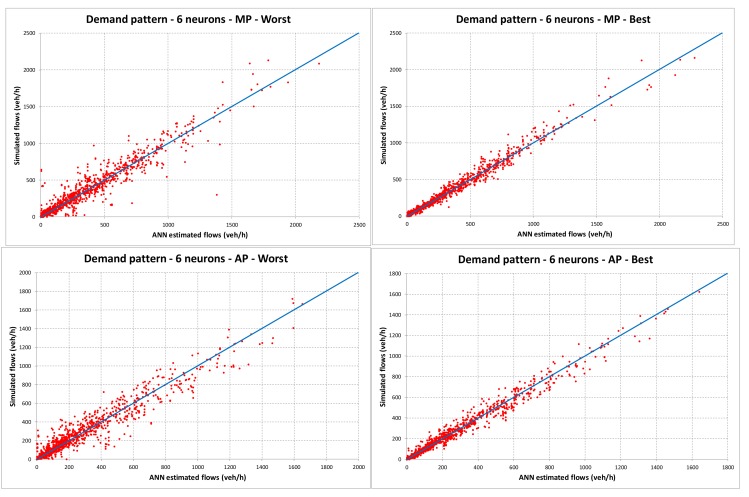

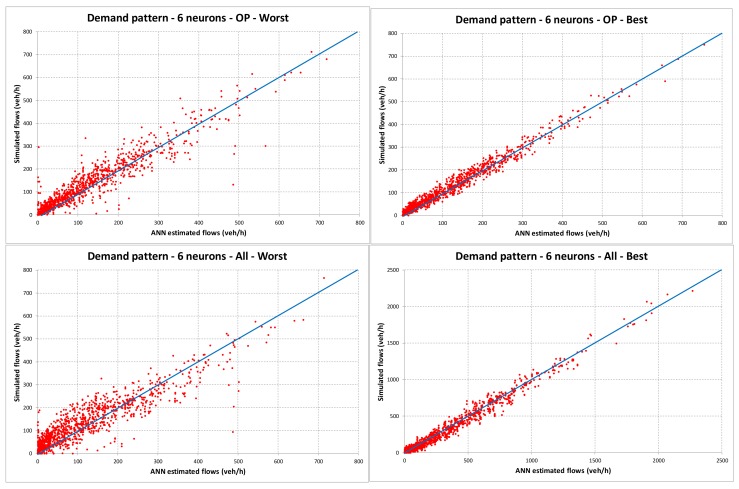
Dispersion diagrams with demand pattern for the ANNs with six neurons (MP: morning peak-hour; AP: afternoon peak-hour; OP: off-peak-hour; All: all periods).

In Figure 3 the dispersion diagrams of worst and best cases of the ANNs trained without considering the demand pattern are reported. In contrast, Figure 4 reports the same diagrams for the ANNs trained considering the demand pattern. All figures refer to ANNs with six neurons, which are the ones that give better results in terms of *RMSE%* and *R*^2^.

The results reported in Table 2 and Figure 4 underline a very good ability of ANNs to estimate the traffic flows on unmonitored links if we consider the demand patterns. Indeed, the values of *RMSE%* are very low and the values of *R*^2^ are also close to 1 in the worst cases. Without considering the demand patterns (see Table 2 and Figure 3), the results can, however, be considered acceptable for this kind of problem, even if the errors are more significant. Indeed, traffic simulation models are affected by some inevitable errors due to (i) the features of road links (travel time, speed, capacity, etc.) that cannot be modelled with high precision and some of them (travel time and speed) depend on flows with functions that have to be correctly calibrated; (ii) the road traffic flows, which also influence the link performances, are always affected by stochastic variations; and (iii) the real origins and destinations of trips are scattered in the territory (the possible points are theoretically infinite) while the model must concentrate them only in some specific points (centroids). For the abovementioned reasons, usually, transportation engineering models with *R*^2^ ≥ 0.75 are considered entirely acceptable. Moreover, it can be noted that the ANNs with six neurons almost always have the best performances.

These results lead to some practical indications for implementing the proposed approach to real cases: (a) to consider explicitly the demand patterns is necessary for obtaining a very good accuracy but, if the estimations of the OD matrices are not available, the results, without considering the demand patterns, can be considered acceptable; (b) ANNs with few neurons (6 or 10) showed the best performances; therefore, it is not necessary to build and train ANNs with many neurons.

## 5. Conclusions

In this paper, the spatial extension of road traffic data from some monitored links to other links is studied and an approach based on artificial neural networks is proposed. Indeed, while in the literature numerous studies can be found on short-term forecasting, the spatial extension of such data has been only marginally studied. The spatial extension may allow for reducing the number of sensors in a monitoring system, controlling the same number of links.

A method based on traffic simulation is proposed for generating the training datasets and the proposed approach is tested on a real-scale network. Two different methods for generating the OD matrices necessary for generating the datasets are adopted: considering, or not, the travel demand patterns; in the first case, the proposed approach can give very good results, while in the second case the results can be considered, however, acceptable.

Future research will be addressed to test other real-scale networks, multi-layer ANNs, and different transfer functions, to verify the improvements that can be obtained in OD matrix correction procedures adding the estimated traffic flows to the surveyed ones, to optimise the location of sensors, and to identify the unmonitored links that are better correlated to the monitored ones, considering only these links as the output of the problem.

## Figures and Tables

**Table 1:** Trained ANNs.

Demand Pattern	Period	Neurons
No	All	6
		10
		20
		50
Yes	MP	6
		10
		20
		50
	AP	6
		10
		20
		50
	OP	6
		10
		20
		50
	All	6
		10
		20
		50

**Table 2:** Forecast error measures.

	Neurons	6		10		20		50	
		Best Case	Worst Case	Best Case	Worst Case	Best Case	Worst Case	Best Case	Worst Case
	**MSE**								
Without demand pattern	All periods	4068.57	2940.26	5750.85	8618.68	8398.84	4395.26	10,902.83	3817.52
With demand pattern	MP	2745.55	12,187.61	2711.22	13,885.76	2794.76	12,428.60	3125.40	13,403.28
	AP	1837.69	6275.93	1788.36	8857.49	2253.55	8686.23	2723.28	8990.26
	OP	352.64	1577.61	319.70	1606.36	312.06	1436.70	487.15	2332.71
	All periods	3273.54	2638.57	2487.71	1855.52	3073.77	2360.71	2842.60	1961.30
	**RMSE**								
Without demand pattern	All periods	63.79	54.22	92.84	75.83	91.65	66.30	104.42	61.79
With demand pattern	MP	52.40	110.40	52.07	117.84	52.87	111.48	55.91	115.77
	AP	42.87	79.22	42.29	94.11	47.47	93.20	52.19	94.82
	OP	18.78	39.72	17.88	40.08	17.67	37.90	22.07	48.30
	All periods	57.21	51.37	49.88	43.08	55.44	48.59	53.32	44.29
	***RMSE%***								
Without demand pattern	All periods	0.00016	0.00034	0.00022	0.00044	0.00023	0.00040	0.00027	0.00038
With demand pattern	MP	0.00013	0.00027	0.00013	0.00029	0.00013	0.00028	0.00014	0.00029
	AP	0.00014	0.00025	0.00014	0.00030	0.00015	0.00030	0.00017	0.00031
	OP	0.00013	0.00027	0.00013	0.00028	0.00013	0.00027	0.00016	0.00034
	All periods	0.00014	0.00033	0.00013	0.00029	0.00013	0.00032	0.00013	0.00030
	***R*^2^**								
Without demand pattern	All periods	0.966	0.786	0.946	0.668	0.931	0.702	0.912	0.762
With demand pattern	MP	0.978	0.908	0.978	0.895	0.977	0.897	0.973	0.890
	AP	0.975	0.925	0.976	0.895	0.971	0.893	0.963	0.906
	OP	0.975	0.894	0.976	0.889	0.976	0.902	0.966	0.846
	All periods	0.975	0.831	0.979	0.877	0.977	0.847	0.978	0.871

**Table 3:** Computing times.

	Neurons	6		10		20		50	
		Epochs	Time	Epochs	Time	Epochs	Time	Epochs	Time
	**MSE**								
Without demand pattern	All periods	447	16′′	465	17′′	544	22′′	489	26′′
With demand pattern	MP	406	17′′	196	8′′	322	14′′	387	21′′
	AP	185	8′′	146	6′′	201	9′′	216	16′′
	OP	197	8′′	300	13′′	583	25′′	596	30′′
	All periods	1000	1′11′′	1000	1′13′′	1000	1′16′′	1000	1′37′′
